# Mechanosensing in macrophages and dendritic cells in steady-state and disease

**DOI:** 10.3389/fcell.2022.1044729

**Published:** 2022-11-17

**Authors:** Megan Lee, Huixun Du, Daniel A. Winer, Xavier Clemente-Casares, Sue Tsai

**Affiliations:** ^1^ Department of Medical Microbiology and Immunology, Faculty of Medicine and Dentistry, University of Alberta, Edmonton, AB, Canada; ^2^ Buck Institute for Research on Aging, Leonard Davis School of Gerontology, University of Southern California, Los Angeles, CA, United States; ^3^ Division of Cellular and Molecular Biology, Diabetes Research Group, Toronto General Hospital Research Institute (TGHRI), University Health Network, Toronto, ON, Canada; ^4^ Department of Immunology, University of Toronto, Toronto, ON, Canada; ^5^ Department of Laboratory Medicine and Pathobiology, University of Toronto, Toronto, ON, Canada; ^6^ Department of Pathology, University Health Network, Toronto, ON, Canada; ^7^ Buck Institute for Research on Aging, Novato, CA, United States; ^8^ Cancer Research Institute of Northern Alberta, University of Alberta, Edmonton, AB, Canada; ^9^ Li Ka Shing Institute of Virology, University of Alberta, Edmonton, AB, Canada

**Keywords:** mechanotransduction, macrophages, dendritic cells, substrate stiffness, integrins, Hippo signalling, Piezo1, TRPV4

## Abstract

Macrophages and dendritic cells are myeloid cells that play critical roles in immune responses. Macrophages help to maintain homeostasis through tissue regeneration and the clearance of dead cells, but also mediate inflammatory processes against invading pathogens. As the most potent antigen-presenting cells, dendritic cells are important in connecting innate to adaptive immune responses *via* activation of T cells, and inducing tolerance under physiological conditions. While it is known that macrophages and dendritic cells respond to biochemical cues in the microenvironment, the role of extracellular mechanical stimuli is becoming increasingly apparent. Immune cell mechanotransduction is an emerging field, where accumulating evidence suggests a role for extracellular physical cues coming from tissue stiffness in promoting immune cell recruitment, activation, metabolism and inflammatory function. Additionally, many diseases such as pulmonary fibrosis, cardiovascular disease, cancer, and cirrhosis are associated with changes to the tissue biophysical environment. This review will discuss current knowledge about the effects of biophysical cues including matrix stiffness, topography, and mechanical forces on macrophage and dendritic cell behavior under steady-state and pathophysiological conditions. In addition, we will also provide insight on molecular mediators and signaling pathways important in macrophage and dendritic cell mechanotransduction.

## Introduction

Mechanotransduction is the process by which cells convert extracellular mechanical and physical cues into intracellular biochemical responses (Martino et al., 2018). Immune cell mechanotransduction is an emerging field, where increasing evidence points to a role for physical cues coming from tissue stiffness in promoting immune cell recruitment, activation, and inflammatory function (Shaheen et al., 2017; Oakes et al., 2009; Adlerz et al., 2016; O'Connor et al., 2012; Chakraborty et al., 2021). This concept applies under steady-state conditions; however, it can be more noticeable during pathological processes that alter tissue stiffness. Throughout their lifetime, immune cells will encounter various mechanical forces and tensions. During fetal development, the first primitive hematopoietic cells are derived from the yolk sac (Moore and Metcalf, 1970), followed by a second wave of hematopoiesis stemming from the fetal liver (Ema and Nakauchi, 2000), and spleen (Christensen et al., 2004), leading to the production of many tissue resident immune cells. Postnatally, nearly all immune cells originate in the bone marrow as hematopoietic stem cells and differentiate through myeloid or lymphoid cell lineages (Sawai et al., 2016). As these cells develop and egress from the bone marrow, they circulate the body *via* the blood and lymphatic system, and traffic through different organs. During this migration, they can face diverse physiological conditions in tissues across the body exhibiting a broad range of stiffness (Table 1).

**TABLE 1:** Elastic moduli of tissues.

Elastic modulus	Tissue	References
<1 kPa	Soft mucosa, brain (0.05–0.5 kPa)	Flanagan et al. (2002)
2–5 kPa	Adipose tissue, lymph nodes	Samani et al. (2003)
0–12 kPa	Lung	McGee et al. (2008)
12 kPa	Cardiomyocytes, skeletal muscle	Engler et al. (2004)
3–16 kPa	Spleen, endothelium	(Arda et al., 2011; Pawluś et al., 2016; Le Master et al., 2018)
5–27 kPa	Bone marrow	Jansen et al. (2015)
40 kPa	Inflamed lymph nodes, high-grade invasive ductal carcinoma tumour (breast cancer)	(Samani et al., 2007; Meng et al., 2020)
20–50 kPa	Cirrhotic liver	Umut Ozcan et al. (2011)
>50 kPa	Cartilage, skin, bone	(Tilleman et al., 2004; Liang and Boppart, 2010; Choi et al., 2013)
35–70 kPa	Fibrotic scar in cardiac tissue	Engler et al. (2008)

**FIGURE 1:**
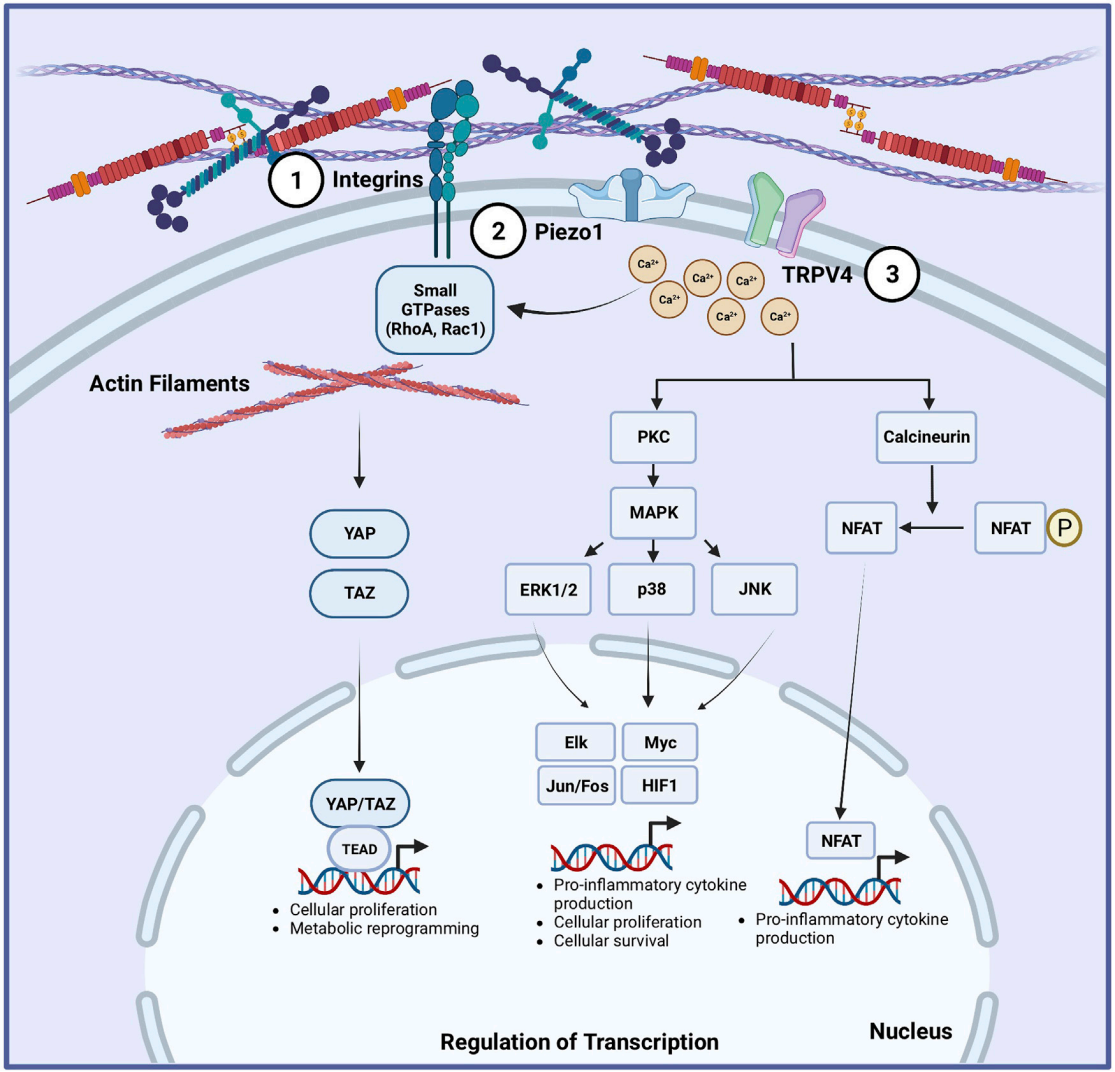
Proposed mechanotransduction pathways in macrophages and dendritic cells. Multiple mechanisms are implicated to translate mechanical cues into cellular responses in macrophages and dendritic cells (DCs) (Martino et al., 2018). Integrins can sense extracellular matrix stiffness changes, which can translate into activation of small GTPases and subsequent modification of the subcellular organization and structure of F-actin filaments. This is linked to activation of the Hippo signaling pathway by increasing YAP/TAZ activation, leading to the upregulation of genes involved in glycolysis, amino acid metabolism, cellular proliferation and cell survival. Mechanosensitive ion channels are expressed in macrophages and DCs, including Piezo1 (Shaheen et al., 2017) in both, and TRPV4 (Oakes et al., 2009) in macrophages. Physical forces change the tension in the plasma membrane of cells, causing the ion channels to open. Piezo1 and TRPV4 opening allows the entry of extracellular Ca^2+^ ions into the cells. The influx of Ca^2+^ ions can activate the protein kinase C (PKC)/mitogen-activated protein kinase (MAPK) pathway or calcineurin/NF-κb pathway, leading to the upregulation of transcriptional programs that can increase cellular proliferation, differentiation, and inflammatory responses.

Under steady-state, these changes in stiffness can be sensed by immune cells through different mechanosensors and induce context-dependent effects in cell development and function (Figure 1). Indeed, accumulating evidence has shown that substrate stiffness is a critical determinant of innate immune responses (Blakney et al., 2012; McWhorter et al., 2015; Mennens et al., 2017; Chakraborty et al., 2021). In this review, we seek to highlight the importance of mechanosensing under steady-state conditions in tissue-resident antigen presenting cells (APCs), specifically macrophages and dendritic cells (DCs). We describe the mechanotransduction pathways and molecular mediators that regulate macrophage and DC behaviour and function, and the underlying metabolic states. We next outline recent data focusing on how mechanotransduction impacts DCs and macrophages in pathophysiological states. Studies examining the impact of substrate stiffness utilize hydrogel substrates to mimic physiological ranges of tissue stiffnesses *in vitro* (Table 1)*.* We refer to compliant substrates as having elastic moduli of <50 kPa, medium stiffness substrates as 50–100 kPa, while high stiffness substrates as being ≥100 kPa.

## The role of mechanical stimuli on macrophages

Macrophages are innate immune cells that play an important role in inflammatory processes by secreting pro-inflammatory cytokines in response to pathogens or damaged tissue. They can also engulf pathogens *via* phagocytosis and act as APCs, helping to bridge innate and adaptive immune responses (Murray and Wynn, 2011). In addition, they are critical for maintaining homeostasis by acting as sentinels and clearing cellular debris and promoting tissue repair (Wynn and Vannella, 2016). During development, progenitors from the yolk sac and fetal liver give rise to tissue-resident macrophages, such as alveolar macrophages in the lung, microglia in the central nervous system, Kupffer cells in the liver, and red pulp macrophages in the spleen (Yona et al., 2013). Tissue-resident macrophages persist through adult life as a self-maintaining population and are involved in homeostatic and organ-specific functions (Davies et al., 2013). Following birth, bone marrow hematopoietic stem cells (HSCs) become the main source of blood monocytes, which not only replenish tissue-resident macrophage populations, but also get recruited following injury or infection (Gordon and Plüddemann, 2017).

Macrophages display remarkable plasticity and can adopt a spectrum of phenotypes by responding to cues in their surrounding environment (Orecchioni et al., 2019). During an inflammatory response, large numbers of circulating monocytes are recruited to the site of tissue injury and can differentiate into an inflammatory classically activated macrophage (commonly referred to as M1 macrophages) by various cytokines such as interferon-γ (IFN-γ) (Dalton et al., 1993). These macrophages produce nitric oxide (NO), reactive oxygen species (ROS), interleukin-1 (IL-1), and tumour necrosis factor (TNF), which have antimicrobial effects (Mills et al., 2000). They also express higher levels of major histocompatibility (MHC) class I and II molecules and have enhanced antigen-presenting capabilities (Dalton et al., 1993). When the inflammatory stimulus is eliminated, factors produced by other immune cells such as pro-resolving lipid mediators and Th2-type cytokines influence macrophages towards an anti-inflammatory phenotype (commonly referred to as M2 macrophages) (Stein et al., 1992). M2 reparative macrophages promote fibrosis and the resolution of inflammatory responses by producing matrix metalloproteinases (MMPs), growth factors, and cytokines such as transforming growth factor beta 1 (TGFβ1) (Mills et al., 2000). In addition, they express molecules such as arginase 1 (ARG1) (Mills et al., 2000), mannose receptor (CD206) (Stein et al., 1992), programmed death ligand 2 (PDL2) (Loke and Allison, 2003) and IL-10 (Gerber and Mosser, 2001) to facilitate the restoration of homeostasis. *In vitro,* the addition of lipopolysaccharide (LPS) with IFN-γ in cultures can polarize macrophages towards an M1-like phenotype, while stimulation with cytokines such as IL-4 and IL-13 polarize them towards a reparative phenotype. Although M1 and M2 macrophage phenotypes were thought to exist as distinct binary cell states, accumulating evidence suggest that macrophages *in vivo* can express markers associated with both phenotypes as exhibit multiple unique functional patterns (Stout et al., 2005; Edwards et al., 2006). Therefore, we henceforth refer to macrophages as being either pro-inflammatory macrophages (PIMs) or pro-resolving macrophages (PRMs). Furthermore, macrophages are now known to express diverse transcriptional profiles when they are exposed to signals present in different tissues and in the context of diseases. The complexity of macrophage activation within the context of disease emphasizes their ability to respond to dynamic changes in environmental stimuli. In addition to responses triggered by ligand-receptor interactions and biochemical cues, recent studies have begun to highlight the importance of extracellular mechanical stimuli on macrophage morphology, polarization, and function.

### Impact of mechanical stimuli on the morphology and migration of macrophages

The morphology of a cell refers to its size, shape, and structure. There is some association between morphology, phenotype and functional activity of cells (Lee et al., 2013; McWhorter et al., 2015; Menzyanova et al., 2019). Recent studies have indicated that the morphological properties of macrophages are influenced by biophysical cues. For example, murine bone marrow-derived macrophages (BMMs) grown on more compliant substrates are rounder and display less spreading compared to BMMs grown on stiffer substrates (Hsieh et al., 2019; Escolano et al., 2021; Haschak et al., 2021). Macrophages derived from human leukemia monocytic THP-1 cells, which is a model for human macrophages, also assume a more aggregated morphology on soft and medium stiffness gels (Sridharan et al., 2019). The surface area of human THP-1 derived macrophages is also significantly different when grown on different substrates, where the cell area increases slightly from a sphere after 18 h on substrates with moduli mimicking healthy arterial stiffness (1–5 kPa), while the area increases almost eight-fold when grown on stiffer substrates (280 kPa-70 GPa) (Adlerz et al., 2016). Furthermore, the percentage of murine BMMs with filopodial extensions increase after being cultured on higher substrate stiffness plates, as BMMs cultured on gels of lower stiffness display few to no filopodial extensions (Patel et al., 2012; Haschak et al., 2021). Alveolar macrophages also exhibit similar flexibility in morphology that adapt to pliant vs. stiff substrates (Féréol et al., 2006). Murine RAW264.7 macrophages or primary human alveolar macrophages cultured on less rigid substrates display a more rounded shape compared to those cultured on a more rigid substrate (Patel et al., 2012). Such morphological adaptations may be highly relevant in the movement of macrophages, which adopt different migration modes in response to environmental constraints (Van Goethem et al., 2010; Vérollet et al., 2011; Travnickova et al., 2021).

Migration is critical for macrophages, as they are highly motile cells and need to migrate within tissues for immune surveillance and respond to pathogens or damage. Macrophages use two main types of motility: amoeboid and mesenchymal. Amoeboid migration is based on flowing and squeezing and is independent of adhesion (Lämmermann et al., 2008), whereas mesenchymal migration is podosome-dependent and involves cell protrusion and adhesion of the leading edge, followed by retraction of the cell rear to achieve movement (Cui et al., 2018). Substrate stiffness is important in dictating the migration mode of macrophages, as human THP-1 derived macrophages demonstrate a fast, podosome-independent migration on more pliant substrates, whereas on stiffer polyacrylamide gels they acquire a slow, podosome-dependent mesenchymal migration mode (Sridharan et al., 2019). In addition, substrate stiffness affects other properties of migration. Migration pathways of human, monocyte-derived macrophages are random regardless of substrate stiffness, but the migration speed is affected (Adlerz et al., 2016). These macrophages move significantly faster on stiffer substrates compared to those on more pliant surfaces, which is consistently seen with murine BMMs (Hsieh et al., 2019). Notably, BMMs migrate further from their starting positions and move significantly faster when cultured on crosslinked fibrin gels compared to when cultured on non-crosslinked fibrin gels, suggesting that mechanical stiffness and the density of the ECM architecture may play a role in the migration of macrophages (Hsieh et al., 2019).

### The impact of mechanical stimuli on macrophage phagocytosis

Macrophages have a high capacity for phagocytosis, which is a process that is crucial for the elimination of foreign materials and apoptotic cells and can be enhanced by opsonization (Acharya et al., 2019). Interestingly, the ability of macrophages to perform their phagocytic functions can be influenced by extracellular mechanical cues. The elasticity, or ability of alveolar macrophages to deform in response to external stress, is significantly higher when cultured on more rigid substrates, which translates into functional differences (Patel et al., 2012). Human alveolar macrophages and murine RAW 264.7 macrophages grown on more rigid substrates have an increased capacity for phagocytosis of bacteria and both unopsonized and IgG opsonized latex beads, which is abrogated when the elasticity of the macrophages is reduced after isotropic biaxial stretch treatment (Patel et al., 2012). The increase in phagocytosis observed could be due to increased migration speed on stiffer substrates (Adlerz et al., 2016), allowing the macrophages to engulf more targets within the same amount of time. Interestingly, human THP-1 derived macrophages exhibit the greatest phagocytic capacity when cultured on medium stiffness gels and followed by softer gels, with the lowest levels of phagocytosis on macrophages cultured on higher stiffness gels (Sridharan et al., 2019). Pliant substrates can favor an anti-inflammatory macrophage phenotype (Blakney et al., 2012; Sridharan et al., 2019), which have higher phagocytic activity compared to inflammatory macrophages (Tierney et al., 2009). However, one study found that the ability of human monocyte-derived macrophages to phagocytose particles was not dependent on substrate stiffness (Adlerz et al., 2016), as the phagocytic events between BMMs grown on more pliant surfaces was comparable to those grown on stiff substrates. The study that showed stiffness-dependent differences in phagocytosis was conducted on substrates of 1.2 kPa (soft) vs. 150 kPa (stiff) (Patel et al., 2012), whereas the study that found no difference utilized substrates of 1–5 kPa vs. 280 kPa (Adlerz et al., 2016). Thus, it is likely that there is a set range of stiffnesses wherein the mechanical signals instruct macrophage function. Multiple factors may interplay to impact phagocytosis, including migration speed as well as the pro-/anti-inflammatory state of macrophages, which are also impacted by substrate stiffness. Thus, more data is needed to tease out the effects of mechanical cues on phagocytosis by macrophages of different tissue origins, as well as the mechanisms driving differences in effects.

### Mechanical stimuli regulate macrophage polarization

In addition to biochemical signals, emerging evidence has highlighted the importance of mechanical stimuli in the modulation of PIM vs PRM activation. However, the effects of stiffness on macrophage polarization are inconsistent across studies.

There are multiple studies that show macrophages adopt a stronger pro-inflammatory phenotype on stiffer gels (Blakney et al., 2012; Previtera and Sengupta, 2015; Sridharan et al., 2019; Haschak et al., 2021). After the addition of PIM polarizing cytokines, naive murine BMMs that were cultured on stiffer gels showed stronger responses, as the expression of PIM-associated protein inducible nitric oxide synthase (iNOS) and the nitrite concentration in the cell culture media supernatants were significantly higher (Haschak et al., 2021). mRNA expression of pro-inflammatory markers *Il1β*, *Mcp1*, *iNOS*, *Il6*, and *Tnfα* were also markedly upregulated when murine BMMs were cultured on stiffer hydrogels (Dutta et al., 2020). Whether murine BMMs were unstimulated or stimulated with LPS, the levels of TNF-α, interleukin one beta (IL-1β), and NO increased in culture supernatants as substrate stiffness increased (Previtera and Sengupta, 2015). Stimulation with IFN-γ and LPS of murine BMMs grown on high stiffness polyacrylamide gels secreted the highest levels of TNF-α and IL-6 compared to BMMs grown on medium and softer gels (Blakney et al., 2012), with CCL20 also being expressed significantly higher (Blakney et al., 2012; Sridharan et al., 2019). Similarly, IL-6 secretion (Sridharan et al., 2019) and iNOS expression (Atcha et al., 2021a) increased when murine BMMs were stimulated with LPS on higher stiffness substrates. Not all studies observed increased production of TNF-α nor IL-6 by unstimulated human promonocytic THP-1 cells cultured on stiffer gels (Sridharan et al., 2019), but pro-inflammatory markers CXCL11 and CCL20 were upregulated (Sridharan et al., 2019).

Additionally, upon PRM induction by culturing human THP-1 derived macrophages with cytokines IL-4 and IL-13, the production of IL-10 is significantly higher on medium and soft stiffness gels, while on stiff polyacrylamide gels, the production of IL-10 is negligible, suggesting that soft and medium stiffness gels enhance anti-inflammatory phenotypes (Sridharan et al., 2019), further supporting the tenet that higher stiffness promotes a pro-inflammatory phenotype. Furthermore, the ability of naive murine BMMs to polarize to PRMs in response to IL-4 was inversely correlated with substrate stiffness, as the activity levels of arginase-1, which is upregulated in murine PRMs (Mills, 2012; Yang and Ming, 2014), was significantly decreased on stiffer substrates (Haschak et al., 2021).

While the majority of studies supported a proinflammatory effect of stiff substrate conditions, a subset of studies reported the opposite. In some studies, culturing murine macrophages on lower substrate stiffness promoted CD86 expression on the cell surface and production of ROS, IL-1β (Joshi et al., 2022) and TNF-α (Chen et al., 2020). In contrast, macrophages grown on medium stiffness gels expressed more CD206, produced less ROS, and secreted more IL-4 and TGF-β compared to macrophages grown on the pliant gels (Chen et al., 2020). Similarly, *Tnf-α* gene expression and/or TNF-α and IL-6 production were significantly higher in LPS-primed murine BMMs grown on more compliant hydrogels, with *Il6* and *Ilb* following similar trends (Escolano et al., 2021). Differentiated human promonocytic THP-1 cells that were attached on interpenetrating polymer network coatings with lower elastic moduli secreted significantly higher levels of TNF-α compared to THP-1 cells attached on higher moduli coatings (Irwin et al., 2008). *In vivo*, two studies showed that stiff substrate conditions and interstitial flow promoted PRM macrophage polarization (Li et al., 2018; Chen et al., 2020).

Altogether, the majority of *in vitro* studies shows positive correlation between PIM polarization and substrate stiffness, with some exceptions (summarized in Table 2). The inconsistencies in the above studies could arise from the variation of culture conditions such as duration, the stiffness range, the source and concentration of differentiation cytokines, the adhesive ligand and the matrix composition, the activation stimuli, and the type of macrophages across the different studies. Studies also utilize a variety of substrate stiffness ranges and define soft and stiff substrates differently. Although we have described compliant substrates as having elastic moduli of <50 kPa, medium stiffness substrates as 50–100 kPa, and high stiffness substrates as being ≥100 kPa based on stiffnesses seen in human tissues, these definitions still cover a wide range of stiffnesses. Standardization and clear definitions on soft *versus* stiff substrates could help reduce the observed discrepancies. In addition, many of the studies rely on the use of *in vitro* culture systems to generate macrophages, which may not accurately reflect the macrophages found *in vivo*. However, *in vivo* studies also have the challenge that macrophages come from a variety of sources and have differences in ontology. Tissue-resident macrophages and monocyte-derived macrophages are highly heterogeneous and could each respond differently to mechanical cues. Further research is needed to elucidate how each of these factors, in conjunction with substrate stiffness, regulates the behaviour of specific types of macrophages and their ability to modulate inflammatory responses.

**TABLE 2:** Summary of effect of mechanical stimuli on the polarization of macrophages.

Macrophage type	Substrate stiffness/Material or mechanical stimuli	Culture duration	Activation stimulus	Treatment duration	Impact of high stiffness	References
Murine BMMs	8 kPa or 32 kPa poly-dimethyl-siloxane hydrogels, or tissue culture plastic coated with decellularized cardiac ECM	7 days	IFN-γ (20 ng/ml)	24 h	• ↑ iNOS expression nitrite concentration	Haschak et al. (2021)
			LPS (100 ng/ml)		• ↓ Arginase-1 activity levels	
Murine thioglycolate-induced peritoneal macrophages	1 kPa or 50 kPa collagen-coated polyacrylamide hydrogels	96 h after *i.p.* injection of thioglycolate	IFN-γ (10 ng/ml)	24 h	• *↑ Il1β, Mcp1, iNOS, Il6,* and *Tnfα* mRNA expression	Dutta et al. (2020)
			LPS (100 ng/ml)			
Murine BMMs	0.3 kPa, 1 kPa, 6 kPa, 27 kPa, 47 kPa, 120 kPa, or 230 kPa poly-d-lysine-coated polyacrylamide hydrogels	7 days	Unstimulated	N/A	• ↑ TNF-α, IL-1β, NO in supernatants	Previtera and Sengupta, (2015)
Human Pro-monocyticTHP-1 cells	11 kPa, 88 kPa, or 323 kPa collagen-coated polyacrylamide hydrogels	26 h	Unstimulated	N/A	• ↑ IL-6 secretion CXCL11, CCL20 expression	Sridharan et al. (2019)
Human Pro-monocyticTHP-1 cells	11 kPa, 88 kPa, or 323 kPa collagen-coated polyacrylamide hydrogels	26 h	IL-4 (20 ng/ml) and IL-13 (20 ng/ml)	72 h	• ↓ IL-10 production	Sridharan et al. (2019)
Murine BMMs	1 kPa, 20 kPa, 40 kPa, or 280 kPa fibronectin-conjugated polyacrylamide hydrogels	7 days	IFN-γ (0.3 ng/ml) and LPS (0.3 ng/ml)	1–18 h	• ↑ iNOS expression	Atcha et al. (2021a)
Murine BMMs	130 kPa, 240 kPa, or 840 kPa poly (ethylene glycol) hydrogels modified with RGD	11 days	Unstimulated	N/A	• ↑ TNF-α and IL-6 secretion, ↑ CCL20	Blakney et al. (2012)
Murine BMMs	2.55, 34.88, or 63.53 kPa polyacrylamide hydrogels	3–5 days	Unstimulated	N/A	• ↓ CD86 expression, production of ROS, IL-1β, TNF-α	Chen et al. (2020)
					• ↓ CD206 expression, ROS, ↑ IL-4 and TGF-β	
*In vivo* 6 week old C57BL/6 mice	Subcutaneous implantation of 2.55 kPa, 34.88 kPa, or 63.53 kPa polyacrylamide hydrogels	14 days	N/A	N/A	• ↑ CD68^+^ CD206^+^ macrophages	Chen et al. (2020)
Murine BMMs	0.2 kPa, 14.3 kPa, or 33.1 kPa polyacrylamide hydrogels	6 days	LPS (100 ng/ml)	6 h	• ↓ Tnf-a, Il6, Ilb expression	Escolano et al. (2021)
					• ↓ IL-6 in culture supernatants	
Human Pro-monocytic THP-1 cells	1.4 kPa, 6 kPa, 9.9 kPa, or 348 kPa interpenetrating polymer network (quartz disks with polyacrylamide gels) modified with RGD	3 days	no stimulation	N/A	• ↓TNF-α in culture supernatants	Irwin et al. (2008)

## The role of mechanical stimuli in dendritic cells

DCs are a heterogeneous population of APCs that are important for innate and adaptive responses to infection. As the most potent APCs, DCs help to stimulate antigen-specific T cell responses to eliminate foreign pathogens (Banchereau and Steinman, 1998). In addition to their immunogenic roles, they play a crucial role in maintaining immune tolerance to self-tissues (Banchereau and Steinman, 1998). Based on their transcriptional programming and functional characteristics, these professional APCs can be classified into monocyte-derived DCs (moDCs), cDC1, cDC2, and plasmacytoid DCs (pDCs) (Steinman and Idoyaga, 2010). moDCs develop from monocytes in the circulation upon stimulation and are involved in inflammation and infection (Marzaioli et al., 2020). cDCs can recognize extracellular and intracellular pathogens and present peptides to CD4^+^ and CD8^+^ T cells (Musumeci et al., 2019). pDCs are important for anti-viral responses and can produce large amounts of type I interferons (Swiecki and Colonna, 2015). Similar to macrophages, DCs are exposed to a diverse array of mechanical environments. DCs originate in the bone marrow and migrate towards peripheral tissues through blood circulation (Liu et al., 2009), and can further travel from peripheral tissues towards lymph nodes through the lymphatic system (Liu et al., 2009). How biochemical cues can influence DC maturation and function has been extensively studied (Jonuleit et al., 1997; Waskow et al., 2008; Schaupp et al., 2020), but recent studies have started to focus on the impact that biophysical stimuli have on DC activation (Craig et al., 2008; Mennens et al., 2017; Kang et al., 2021), migration (Mennens et al., 2017; Kang et al., 2021), function (Lewis et al., 2013; Chakraborty et al., 2021), and metabolism (Pearce and Everts, 2015; Chakraborty et al., 2021).

### Mechanical signals in DC activation

DCs can be activated directly by recognition of conserved pathogenic molecules *via* their pattern recognition receptors, and indirectly by inflammatory mediators produced by other cell types that have recognized foreign materials (Cabeza-Cabrerizo et al., 2021). Activation of DCs will lead to the expression of appropriate ‘maturation markers’, including CD80, CD86, CD83, high levels of major histocompatibility (MHC) class I and II molecules, and CD40 (Cabeza-Cabrerizo et al., 2021). Studies have highlighted the impact that extracellular mechanical stimuli can play on the activation and expression of these maturation markers. The development and maturation of DCs are affected by extracellular pressure (Craig et al., 2008). Specifically, the expression of activation markers (CD80, CD86, CD83, CD40) and MHC class II molecules is significantly upregulated on mature moDCs isolated from healthy human donors exposed to elevated pressure in an airtight Lucite box (Craig et al., 2008). Similarly, immature human moDCs showed a significant increase in the expression of CD80, CD86, CD83, and MHC class II molecules when maintained at elevated pressures (Craig et al., 2008). Using a microfluidic channel to mimic inflammatory edema, murine bone marrow-derived DCs (BMDCs) exposed to higher shear stress show increased expression of the activation markers MHC class I and CD86 compared with DCs under static conditions (Kang et al., 2021). In terms of static substrate stiffness effects on DCs, murine BMDCs grown on the stiffer hydrogels display significantly increased expression of CD80 and CD86 compared to those grown on more pliant hydrogels, with a trending increase in MHC class II molecules (Chakraborty et al., 2021). Consistently, CD83 and CD86 expression is significantly higher on moDCs cultured on higher stiffness substrates (Mennens et al., 2017). However, there are no significant differences in MHC class II molecule expression between human moDCs cultured on 2 kPa, 12 kPa, or 50 kPa substrates (Mennens et al., 2017), suggesting that substrate stiffness may not play as significant of a role in influencing the expression of MHC class II molecules. In addition to pressure and substrate stiffness, DCs residing in the interior layers of the arterial wall experience transmural normal forces from blood flow strain arteries, which translates into a cyclic axial strain of the vessel wall layers (Vanepps and Vorp, 2007). Murine BMDCs cultured *in vitro* on ECM proteins (laminin, collagen, fibrinogen) that are exposed to cyclic strain increased expression of co-stimulatory molecules CD86 and CD40, and MHC class II molecules compared to BMDCs not exposed to any strain (Lewis et al., 2013).

### Impact of mechanical stimuli on the migration of DCs

The directed migration of DCs is essential during inflammatory responses, as they are professional APCs that transport antigens from the periphery to draining lymph nodes to help initiate adaptive immune responses (Hampton and Chtanova, 2019). The migration of mature DCs from peripheral tissues to lymph nodes is regulated by the CC-chemokine receptor 7 (CCR7), which senses levels of chemokine (C-C motif) ligand 19 (CCL19) and CCL21, causing DCs to follow the concentration gradient leading towards lymphatic vessels (Förster et al., 1999). CCR7 expression is lower on mature human moDCs conditioned on 12 kPa substrates compared to those on 2 and 50 kPa, which translates into a significantly lower level of CCL21-mediated migration (Mennens et al., 2017). Additionally, the formation of podosomes, which are important for DC adhesive and migratory behaviour, is significantly decreased in moDCs cultured on 12 kPa and 50 kPa compared to those conditioned on 2 kPa (Mennens et al., 2017). Although there is an increase in the proportion of DCs that migrate at 2 kPa, migration velocity is comparable between moDCs conditioned on the lower and higher substrate stiffnesses (Mennens et al., 2017). Similarly, murine BMDCs exposed to differing levels of shear stress ranging from 0.2–0.6 dyne/cm^2^ did not significantly differ in their migration speed (Kang et al., 2021). However, increased shear stress potentiated their migratory abilities, as the BMDCs under higher shear stress followed more straightforward trajectories and demonstrated improved directness (Kang et al., 2021). Therefore, these findings suggest that biomechanical cues may not affect DC migration velocity, but can impact the ability of DCs to migrate effectively.

### Mechanical stimuli regulate DC effector function

Upon activation, DCs can perform immunogenic functions that are important for the clearance of pathogens, including the production and secretion of pro-inflammatory cytokines. When studying the effects of substrate stiffness on DC cytokine production, murine BMDCs that were cultured on higher substrate stiffnesses produced higher concentrations of TNF-α, IL-1α, IL-1β, IL-6, IL-12, monocyte chemoattractant protein-1 (MCP-1), and macrophage inflammatory protein-2 (MIP-2) in response to LPS stimulation (Chakraborty et al., 2021). Similarly, in response to pressure stimuli, the production of pro-inflammatory cytokines TNF-α, IL-6, and IFN-γ was significantly increased when the human moDCs were exposed to higher pressures (Craig et al., 2009). Interestingly, the cytokine production of murine BMDCs under cyclic axial strain compared to non-stretched cells was similar (Lewis et al., 2013), suggesting that only certain types of mechanical stimuli may regulate the production of cytokines by DCs.

In addition to cytokine production, another key function of DCs is to act as professional APCs. For the uptake of antigens, DCs express various types of pattern recognition receptors, including the class of C-type lectin receptors (CLRs), which recognize carbohydrate structures (Cerboni et al., 2013). Substrate stiffness impacts the expression of CLRs on DCs, where human moDCs cultured on 2 kPa compared to 12 kPa substrates have 3-fold higher levels of CLRs, but the expression is intermediate on moDCs conditioned on 50 kPa gels (Mennens et al., 2017). This translates into functional differences, as moDCs conditioned on 2 kPa are more capable of C-type lectin-dependent antigen internalization and took up 1.5-2 fold more ovalbumin compared to those conditioned on 12 and 50 kPa (Mennens et al., 2017). Conversely, other studies have found that the phagocytic capability of murine BMDCs was enhanced when they were cultured on 50 kPa hydrogels compared to 2 kPa hydrogels (Chakraborty et al., 2021).

Interactions between DCs and CD4^+^ or CD8^+^ T cells are also impacted by environmental mechanical cues. When examining the effects of cyclical axial strain, murine BMDCs under 3% cyclical strain are more effective at inducing CD4^+^ T cell proliferation (Lewis et al., 2013). Using an E.G7 tumour model, we showed that murine BMDCs grown on stiffer substrates and subsequently injected into tumour-implanted mice induced tumour-killing at a faster rate compared to BMDCs grown on more pliant hydrogels (Chakraborty et al., 2021). The increased efficacy in tumour clearance in mice immunized with BMDCs grown on 50 kPa hydrogels was also associated with an increase in the frequency of effector memory T cells in the CD4^+^ and CD8^+^ T cells compartments (Chakraborty et al., 2021). Another study showed that T cells stimulated by murine BMDCs with increased cytoskeletal stiffness require a lower antigen concentration for activation than do T cells stimulated by BMDCs with softer cytoskeletal stiffness, indicating that DC cytoskeletal stiffness may promote T cell priming (Blumenthal et al., 2020). Altogether, this suggests that exposure to stiff extracellular matrices endows DCs with an enhanced ability to interact with and activate CD4^+^ and CD8^+^ T cells. In Table 3, we compile the studies performed to date supporting the overall stimulatory impact of substrate stiffness on APC-mediated T cell activation.

**TABLE 3:** Summary of effect of mechanical stimuli on the effector function of DCs.

Cells	Substrate stiffness/Material/Mechanical stimuli	Culture duration	Activation stimulus	Treatment duration	Results with high stiffness/Mechanical force	References
Murine BMDCs	2 kPa or 50 kPa poly-dimethyl-siloxane hydrogels	9 days	LPS (100 ng/ml)	24 h	• ↑ TNF-α, IL-1α, IL-1b, IL-6, IL-12, MCP-1	Chakraborty et al. (2021)
					• ↑ phagocytic capacity	
					• ↑ anti-tumour immunity	
Human monocytic DCs	Ambient or 40 mmHg pressure in a Lucite box	6 days	LPS (100 ng/ml), IL-1β (10 ng/ml), IL-6 (1,000 U/mL), TNF-α (10 ng/ml)	24 h	• ↑ TNF-α, IL-6, IFN-y in supernatants	Craig et al. (2009)
Murine BMDCs	3% or 10% cyclic axial strain at 1 Hz with Flexcell-4000	10 days	Cyclic strain	1 h	• No difference in cytokine production under various cyclical axial strain	Lewis et al. (2013)
					• 3% cyclical strain most effective at inducing CD4^+^ T cell proliferation	
Human monocytic DCs	2 kPa, 12 kPa, or 50 kPa polyacrlyamide hydrogels coated with human fibronectin	6 days	Unstimulated	N/A	• ↓ CLR expression, ↓ C-type lectin-dependent antigen internalization	Mennens et al. (2017)
					• T cell activation capacity similar between different stiffnesses	

## Mechanotransduction pathways in macrophages and dendritic cells

**FIGURE 2:**
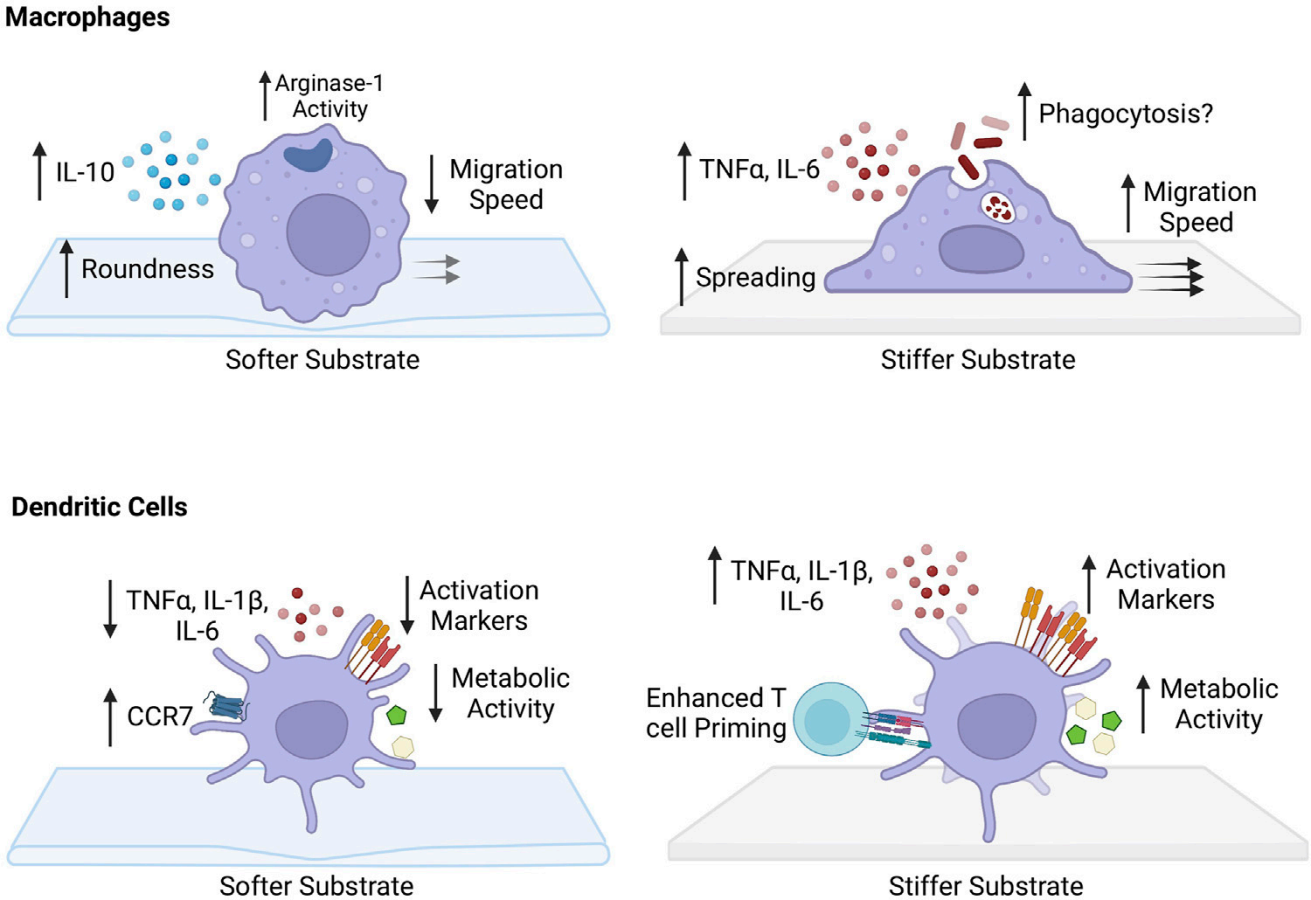
Impact of substrate stiffness on macrophages and dendritic cells. Softer substrates increase the roundness of macrophages. There is an increase PRM-like phenotype with an increase in IL-10 production and arginase-1 activity following IL-4 stimulation. Migration speed of macrophages grown on more pliant substrates is decreased and podosome-dependent. Macrophages cultured on stiffer substrates have a stretched morphology, with increased pro-inflammatory phenotype and secretion of pro-inflammatory cytokines TNF-α and IL-6. Phagocytosis capability has been suggested to increase, but still remains unclear. They acquire a fast, podosome-independent mode of migration after growth on stiffer surfaces. Dendritic cells (DCs) grown on softer substrates appear to have enhanced migratory capacity with increased expression of chemokine receptor 7 (CCR7). There is a decreased ability to produce pro-inflammatory cytokines TNF-α, IL-1β, and IL-6, and decrease in activation state. Metabolically, there is a decrease in glycolytic capacity. DCs cultured on stiffer substrates have greater ability to prime CD4^+^ and CD8^+^ T cells. They have increased capability to produce TNF-α, IL-1β, and IL-6 and have an enhanced activation state with increased expression of CD80/86 and CD40. Metabolically, they have increased glyocolytic capacity and express more intermediates of glycolysis.

As mechanical stimuli impact macrophage and DC activation and function, it is important to understand the specific mechanosensing pathways used by macrophages and DCs in these settings. In the following section, we discuss the molecular basis of mechanosensing including pathways that have been implicated including integrins, Hippo signalling mediators Yes-associated protein (YAP) and its homologue Transcriptional coactivator with PDZ-binding motif (TAZ), as well as mechanosensitive ion channels Transient Receptor Potential (TRP) of vanilloid subtype TRPV4 and Piezo Type Mechanosensitive Ion Channel Component 1 (PIEZO1) (See Figure 2). We also highlight recent advances in understanding how these pathways integrate environmental stimuli to effect functional changes.

### Integrins and Rho GTPases

Cell adhesion molecules, such as integrins (Tamkun et al., 1986), can sense changes in the stiffness of the extracellular matrix (ECM). Broadly, alterations in ECM stiffness are detected and translated into signals that modulate the subcellular organization and fine structure of cytoskeletal components such as F-actin filaments (Tamkun et al., 1986). In turn, this can regulate intracellular signalling pathways that ultimately impact cellular function. Integrins are adhesion receptors consisting of α and β subunits that participate in mechanotransduction. They bind to ECM and are important for bridging the extracellular environment with the intracellular cytoskeleton (Hynes, 2002). In addition, the activation state of integrins can be controlled by mechanosensitive ion channels such as Piezo1 through the modulation of intracellular calcium levels (Aglialoro et al., 2020).

In macrophages, there is a correlation between the levels of β2 and β3 integrins expressed and both adhesive (Weerasinghe et al., 1998; Yakubenko et al., 2008) and migratory (Féréol et al., 2006) abilities of macrophages, and the inhibition of these integrins has been shown to decrease these activities (Féréol et al., 2006; Yakubenko et al., 2008). Furthermore, integrin subtypes are critical for phagocytosis (Blystone et al., 1994), as antibodies to alpha v beta three blocked phagocytosis of fibronectin-opsonized beads completely. Inflammatory activation of macrophages can also be negatively regulated by integrin signalling, as macrophages from mice that were deficient in αM integrin showed increased TNF-α production following LPS stimulation compared to macrophages from wild-type mice (Han et al., 2010). In addition, integrin engagement can lead to the formation of podosomes in macrophages, which are adhesive structures important for macrophage migration (Meli et al., 2019). These podosomes are also connected to the intracellular cytoskeleton, therefore implicating them as potential mechanosensors (Collin et al., 2008; Van Goethem et al., 2010). Whether podosomes exhibit mechanosensory properties during macrophage migration will require further investigation.

Regulators of the cytoskeletal network downstream of integrin signalling, including Rho GTPases, have been put forth as mediators connecting mechanical stimuli to DC function and homeostasis. Integrins act to retain cDC2s in blood-exposed regions of the spleen (Liu et al., 2020) and may participate in sensing shear stress. Ras homolog family member A (RhoA) has been ascribed a critical role in regulating DC homeostasis (Li et al., 2015). In the context of mechanosensing, RhoA and its associated Rho GTPase-activating protein (RhoGAP) regulate actin cytoskeleton rearrangement in response to mechanical forces (Lessey et al., 2012), and also contributes to reciprocal activation of integrin (through inside-out signaling (Laudanna et al., 1996)). In particular, the RhoGAP/RhoA/ROCK signalling pathway in DCs was found to be important in regulating directed migration of mature DCs to secondary lymphoid organs, as BMDCs that lacked effective RhoGAP signalling had exhibited significantly lower directness in response to CCL21 compared to BMDCs from wildtype mice (Xu et al., 2014). Cytoskeletal rearrangements downstream of integrin signaling are also critical in processes such as immune synapse formation between DCs and T cells; this area of research has been extensively reviewed elsewhere (Rodríguez-Fernández and Criado-García, 2021).

### Hippo signalling mediators: MST1/2 and YAP/TAZ

The Hippo signalling pathway is an evolutionarily conserved kinase cascade that is important for regulating cell survival, proliferation, and differentiation (Misra and Irvine, 2018). Upon activation, the core components of the pathway, mammalian STE20-like kinase 1 (MST1) and MST2, work alongside the scaffold protein SAV1 to phosphorylate and activate large tumor suppressor 1 (LATS1) and LATS2 kinases (Chan et al., 2005). In turn, LATS1/2 cooperate with the co-factor Mps1-binder 1 (MOB-1) (Hergovich et al., 2006) to phosphorylate key downstream mediators of the Hippo pathway, YAP (Zhao et al., 2007) and its homologue TAZ (Lei et al., 2008). Phosphorylation of YAP and TAZ leads to their inhibition, as they are sequestered in the cytoplasm by 14.3.3 binding proteins or targeted for proteasomal degradation (Zhao et al., 2007). When the Hippo pathway is switched off, MST1/2 and LATS1/2 remain dephosphorylated, preventing the inhibition of YAP and TAZ (Zhao et al., 2007; Lei et al., 2008). Thus, YAP and TAZ can translocate to the nucleus and bind to TEA domain transcription factors (Vassilev et al., 2001; Mahoney et al., 2005). The activity of YAP and TAZ can be regulated through mechanical inputs sensed by integrins. For example, the activation of YAP and TAZ by focal complex formation is linked to the activation of F-actin modulators such as GTPase Rac1 and its effector p-21 activated kinase (PAK), and Rho guanidine exchange factor β-PIX (Sabra et al., 2017). In turn, F-actin levels can modulate LATS1/2 kinase activity and consequently YAP and TAZ signalling (Aragona et al., 2013) or can facilitate the nuclear entry of YAP and TAZ by directly impacting the mechanics of the nucleus through nesprin and Sad1-UNC-84 (SUN) complexes (Driscoll et al., 2015). As a result, there is an upregulation of genes involved in metabolic programs such as glycolysis (Wang et al., 2015) and amino acid metabolism (Yang et al., 2018), cellular proliferation (Koo et al., 2020), and cell survival (Pavel et al., 2018).

In macrophages, YAP/TAZ has been found to play a role in PRM polarization within tumours (Huang et al., 2017; Yang et al., 2020), and also during TGFβ1-induced fibrosis (Feng et al., 2018). Also, the expression of YAP/TAZ is increased in macrophages with a pro-inflammatory phenotype, with the activation of YAP enhancing pro-inflammatory responses, while the genetic deletion of YAP and TAZ dampening pro-inflammatory responses and enhancing reparative responses (Mia et al., 2020). In the setting of myocardial infarction, altered macrophage polarization leads to a reduction in cardiac fibrosis and hypertrophy and an improvement in overall cardiac function (Mia et al., 2020). In the context of inflammatory bowel disease (IBD), YAP-deficient mice exhibited higher numbers of pro-resolving polarized macrophages in colonic tissue, which helped protect mice from IBD (Zhou et al., 2019). Concerning mechanical stiffness, adhesion of macrophages to soft hydrogels reduces inflammation when compared to adhesion on stiff materials, and is associated with reduced YAP expression and nuclear localization (Meli et al., 2020). Furthermore, the depletion of YAP inhibits macrophage inflammation, whereas overexpression of active YAP increases inflammation, shown by differences in pro-inflammatory cytokine secretion (Meli et al., 2020). When cultured on various hydrogel materials (collagen, Matrigel, and polyethylene glycol (PEG)), macrophages secreted less TNF-α compared to cells on polystyrene controls (Meli et al., 2020). Additionally, upstream modulators of YAP/TAZ in the Hippo signalling cascade, MST1/2, have been shown to play a role in regulating macrophage phenotypes. Specifically, mice with a specific deficiency of MST1/2 in macrophages displayed impaired post-myocardial infarction repair compared to wild-type mice (Liu et al., 2015). Although these recent findings have shed light on the importance of YAP/TAZ signalling in mediating macrophage function and influencing their polarization and phenotype, the exact mechanism by which YAP/TAZ regulates these changes remains uncertain, as many upstream signals can regulate YAP/TAZ expression and activity (Totaro et al., 2018).

Similarly, DCs can sense mechanical stimuli *via* YAP/TAZ, as transcriptomic analysis showed a marked upregulation of TAZ in the BMDCs grown on 50 kPa compared to 2 kPa, which was validated by RT-qPCR (Chakraborty et al., 2021). Additionally, the stiff substrate-induced production of TNF-α was abrogated when the BMDCs were cultured with verteporfin, an inhibitor of YAP/TAZ (Chakraborty et al., 2021). The Hippo signalling pathway was further implicated in DC mechanotransduction, as CD8α^+^ DCs had enrichment of kinases involved in Hippo signalling, including the phosphorylation of Mst1/2, Yap, and Lats1 (Du et al., 2018). DC-specific deletion of Mst1/2 disrupted the homeostasis and function of CD8α^+^ DCs and led to the impaired presentation of cognate peptides to prime CD8^+^ T cells (Du et al., 2018). However, CD8α- DCs deficient in Mst1/2 overall exhibited normal function (Du et al., 2018), suggesting that the role of various mechanosensing pathways may differ for specific subtypes of DCs.

### Mechanosensitive ion channels: PIEZO1 and TRPV4

In addition to integrins, immune cells express mechanosensitive ion channels that modulate cellular activity through the gating of soluble ions. One family of ion channels that are found on the surface of immune cells are the Piezo proteins. PIEZO1 and PIEZO2 channels are found in most mammals and convert mechanical stimulation into biological signals (Parpaite and Coste, 2017). These mechanosensitive channels respond to physical forces such as shear stress, which leads to changes in tension in the plasma membrane, causing them to open. PIEZO1 and PIEZO2 are permeable to monovalent cations including K^+^ and Na^+^, as well as divalent cations such as Ca^2+^ and Mg^2+^ (Parpaite and Coste, 2017). Therefore, the opening of PIEZO channels results in the influx of cations and cell membrane depolarization, which can initiate intracellular Ca^2+^ signalling pathways that can impact the activation states of immune cells (Solis et al., 2019).

PIEZO1 is one mechanically activated cation channel recently found to regulate macrophage biology. Culturing on stiff substrates promoted calcium influx into macrophages in a PIEZO1-dependent manner (Atcha et al., 2021a). Genetic ablation of Piezo1 in macrophages reduced inflammatory potential and enhanced wound healing responses (Atcha et al., 2021a). Furthermore, siRNA knockdown of Piezo1 abrogated any stretch-mediated changes in inflammatory responses, further highlighting the role of PIEZO1 in macrophage mechanosensation (Atcha et al., 2021b). PIEZO1 has also been implicated in bridging DC mechanosensation to function. DCs cultured on lower substrate stiffness conditions produced significantly lower levels of pro-inflammatory cytokines, but with the addition of PIEZO1 agonist Yoda-1, there was a marked upregulation of TNF-α and IL-6 production in the cell culture supernatants of BMDCs grown on softer substrate conditions (Chakraborty et al., 2021). PIEZO1 activation also led to a significant increase in the transcription of glycolytic genes, suggesting that PIEZO1 could also play a role in altering DCs metabolic state (Chakraborty et al., 2021). Additionally, mice with DC-specific deficiency of Piezo1 exhibited a moderately decreased anti-tumour response (Chakraborty et al., 2021; Wang et al., 2022), associated with an increase in the differentiation of T_reg_ and decrease in the generation of T_H_1 cells (Wang et al., 2022). Furthermore, PIEZO1 can potentiate integrin-mediated mechanosensing and adhesion. Activation of Piezo1 leads to the activation of Ca^2+^-mediators Calpain and Protein kinase C (PKC), which in turn increases the activation of integrins (Aglialoro et al., 2020). Overall, these findings suggest that Piezo1 could be important in DC function and metabolism.

Another category of ion channels found on immune cells that can sense mechanical cues includes the Transient receptor potential (TRP) channels. More specifically, TRP channels of the vanilloid subtype such as TRPV1, TRPV2, and TRPV4 have been identified as being sensitive to alterations in membrane stretch, pressure, and shear stress (Baylie and Brayden, 2011). The opening of TRPV channels allows for the entry of extracellular Ca^2+^, which triggers signalling cascades that alter transcription, vesicular transport, and cytoskeletal remodeling (Baylie and Brayden, 2011).

Macrophages express transient receptor potential (TRP) family channels, including TRPV2, TRPV4, TRPC6, and TRPM7, which have been shown to play an important role in macrophage polarization (Dutta et al., 2020; Li et al., 2020), inflammatory activation (Scheraga et al., 2016; Schappe et al., 2018), and phagocytosis (Link et al., 2010; Riazanski et al., 2015; Scheraga et al., 2016). However, TRPV4 channel activity seems to be the only one that is influenced by mechanical stimuli in macrophages (Scheraga et al., 2016). When BMMs are cultured on stiffer substrates, stimulation with LPS led to increases phagocytic capacity and intracellular calcium influx compared to BMMs grown on softer substrates (Scheraga et al., 2016). The effect of stiffer substrates is abrogated when TRPV4 is pharmacologically inhibited or its expression was reduced (Scheraga et al., 2016), and reintroducing TRPV4 expression into TRPV4 KO macrophages restores expression of stiffness-induced PIM markers (Dutta et al., 2020), therefore suggesting that TRPV4 mechanotransduction is important for macrophage function. However, the specific role that a variety of biophysical cues play in influencing channel activity such as mechanical forces and shear stress is not well understood.

### Nuclear sensing pathways

Nuclear sensing pathways can also impact immune cell behaviour and function. Changes in cell shape can be translated by the nucleus into a deformation signal, impacting downstream cellular behaviour. Inner nuclear membrane unfolding can be induced by alterations in cell shape, which in turn leads to myosin II recruitment to the cell cortex, ultimately regulating actin cytoskeleton contractility and cellular behaviour (Venturini et al., 2020). In CD4^+^ T lymphocytes, engagement of the TCR causes the release of Ca^2+^ into the cytoplasm and nucleus, which induces activation of the Actin Related Protein 2/3 complex (Arp2/3) and the polymerization of actin in the nucleus (Tsopoulidis et al., 2019). The rapid formation of an actin filament network in the nucleus in turn regulates cytokine expression.

During cellular migration, mechanical signals may also be transmitted to the nucleus from protruding and retracting cell boundaries *via* the cytoskeletal network. The linker of nucleoskeleton-to-cytoskeleton (LINC) complexes, which comprise nesprin-family proteins connected to SUN proteins, transmits the mechanical signal from the cytoskeleton directly to the nuclear envelope (Alam et al., 2015). Signalling through this complex allows the nucleus to be positioned centrally and allow for transduction of forces along the length of the cell and ensure normal migration (Alam et al., 2015). In addition, cells can monitor their own shape and respond when deformed below a specific height (Lomakin et al., 2020). When compression or confinement of cells is greater than the size of the nucleus, the nuclear envelope unfolds and stretches, triggering release of calcium (Lomakin et al., 2020). This leads to the activation of calcium-dependent cytosolic phospholipase A2 (cPLA2), which catalyzes the formation of arachidonic acid (Lomakin et al., 2020). Arachidonic acid increases the adenosine triphosphatase activity of myosin II, inducing greater contractility and increased motility (Lomakin et al., 2020). Upon confinement using a microfabricated confiner device, immature murine BMDCs DCs demonstrate increased migration speed and demonstrate a migratory cell shape phenotype (Lomakin et al., 2020). However, this is abrogated when cPLA2 and nuclear structural component lamin A is ablated (Lomakin et al., 2020), suggesting this nuclear sensing pathway is important for modulating DC migration.

Extensive work has been done to advance the knowledge of mechanotransduction pathways in immune cells. However, there remains limited studies focusing on the molecular mechanisms by which mechanical stimuli are sensed by macrophages and DCs, and how this translates into functional differences. Therefore, further investigation is required focusing on the specific mechanotransduction pathways influencing macrophage and DC activation, function, and metabolism. Additionally, research on the role of nuclear mechanosensation in macrophages and DCs is limited and requires further work, as well as how they integrate with other mechanotransduction pathways.

## Mechanosensitive metabolic adaptations in macrophages and dendritic cells

Immunometabolism is an emerging area of research, where a growing body of literature has begun to shed light on the importance of metabolic reprogramming in immune cell activation, function, and survival (Jung et al., 2019). Metabolic pathways and programs are tightly regulated, and can strongly influence function and differentiation. Stress due to inadequate intake of proteins and calories has been shown to compromise innate and adaptive immune functions (Ibrahim et al., 2017), while excess in nutrients can promote inflammation and immune dysregulation (Hotamisligil et al., 1993). Although it has been shown that cytokines and factors released from metabolic tissues can regulate immune cell metabolism (Hotamisligil et al., 1993), recent evidence has highlighted how biophysical cues can impact the metabolism of macrophages and DCs.

### Impact of mechanical stimuli on the metabolism of macrophages

Effector functions of macrophages are influenced by metabolic pathways. Traditionally, an elevated level of glycolysis has been associated with LPS-induced-PIM macrophages (Haschemi et al., 2012), as it drives the pentose phosphate pathway (PPP) to boost NADPH necessary for the generation of important pathogen-eliminating agents, such as reactive oxygen species (ROS) and nitric oxide (NO) (Ham et al., 2013). However, some recent research has confirmed that glycolysis is also crucial for IL-4 induced-PRM activation as well (Huang et al., 2016), but unlike PIMs, there is less involvement of the PPP (Haschemi et al., 2012). Additionally, the TCA cycle and mitochondrial oxidative phosphorylation (OXPHOS) are disrupted in PIMs, contributing to an accumulation of citrate and succinate, as well as enhanced fatty acid synthesis, which together support pro-inflammatory secretory phenotypes (Moon et al., 2015). Conversely, an intact TCA cycle, augmented OXPHOS, and fatty acid oxidation are typically associated with anti-inflammatory phenotypes.

Mechanotransduction pathways such as Yes-associated protein (YAP) and Transcriptional coactivator with PDZ-binding motif (TAZ) from the Hippo signaling cascade and ion channel Piezo Type Mechanosensitive Ion Channel Component 1 (PIEZO1) can regulate the metabolic program in many cell types. For example, in response to stiff substrate signals, activated YAP and TAZ translocate to the nucleus and mediate the transcription of genes involved in glucose and amino acid metabolism that are important for macrophage polarization, including *Slc2a3* (glucose transporter 3) (Cosset et al., 2017), *Hk2* (hexokinase 2) and *Pfkfb3* (phosphofructokinase B3) (Zheng et al., 2017) from glycolysis, and *Gls* (glutaminase) (Bertero et al., 2016), *Got1* (glutamic-oxaloacetic transaminase 1) and *Psat1* (phosphoserine aminotransferase 1) (Yang et al., 2018) from glutamine metabolism. In response to hydrostatic pressure, PIEZO1 levels in macrophages are upregulated, followed by PIEZO1 induction of endothelin-1, which stabilizes hypoxia-inducible factor 1α (HIF1α) (Solis et al., 2019) to promote glycolytic metabolism and PPP (Wang et al., 2017). The upregulation in these metabolic pathways due to increased stiffness further supports the idea that increased stiffness promotes pro-inflammatory programs in macrophages. Finally, epigenetic modification is an additional layer of regulation in macrophage activation, particularly histone deacetylase 3 (HDAC3) has been shown to modulate LPS-induced early responses (Jain and Vogel, 2018). HDAC3 can translocate to mitochondria to deacetylate and deactivate the fatty acid oxidation (FAO) enzyme mitochondrial trifunctional enzyme subunit α (HADHA) and then restrict FAO-driven OXPHOS to facilitate pro-inflammatory activation in macrophages (Chi et al., 2020). Given that total HDAC3 levels decrease when physical environments hinder cell spreading, this reduction might be another mechanism by which spatial confinement blunts pro-inflammatory activation (Jain and Vogel, 2018). Thus, more work is needed to understand mechanisms by which mechanical cues dictate macrophage inflammatory function, and one promising avenue of research is to tease out connections linking mechanical cues to metabolic pathways.

### Impact of mechanical stimuli on the metabolism of DCs

The metabolic requirements of inactivated, quiescent DCs are unique from activated DCs, and thus DCs adapt their metabolic programs to optimally support function (Pearce and Everts, 2015). For instance, resting DCs which have relatively few anabolic demands rely on both glycolytic and oxidative metabolic pathways (Pearce and Everts, 2015), whereas the triggering of TLRs stimulates an increase in glycolytic programming to support the anabolic needs of DC activation and maturation (Krawczyk et al., 2010; Everts et al., 2014). Emerging evidence from our lab highlights the impact of mechanical stimuli on DC metabolism and function. By comparing murine BMDCs cultured on pliant (2 kPa) *versus* stiff (50 kPa) hydrogel substrates, as well as plastic substrate, we showed that BMDCs responded to higher stiffness by upregulating glucose metabolism, as reflected by increased glycolysis gene expression, metabolic flux, and glucose uptake (Chakraborty et al., 2021). Furthermore, inhibition of glycolysis with 2-deoxyglucose impaired BMDC’s ability to activate T cells high substrate stiffness (Chakraborty et al., 2021). Such metabolic and functional responses suggest that substrate stiffness can act as an environmental proinflammatory stimuli to promote the antigen presenting function of DCs, through upregulating costimulatory signals in this *in vitro* experiment. Whether similar mechanosensitive metabolic adaptations exist *in vivo* await further investigation, which will shed light on the intricate cross-talks between intracellular metabolic pathways and processes of antigen capture, processing, and presentation in the face of extracellular mechanical signals.

## Mechanotransduction in macrophages and dendritic cells during pathophysiological conditions

Given the accumulating evidence illustrating the importance of mechanical cues on immune function, it is crucial to understand their impact on immune cells under different conditions and diseases, as well as the mechanosensing pathways mediating these effects. This is especially important because the stiffness of tissues can undergo substantial biomechanical alterations and significantly increase during pathophysiological conditions. Numerous studies have shown that biophysical changes in tissues are correlated with disease progression (Ingber, 2003). Specifically, an increase in tissue stiffness has been associated with atherosclerosis (Gotschy et al., 2013; Palombo and Kozakova, 2016), cardiovascular disease (Blacher et al., 1998), inflammatory bowel disease (IBD) (Stewart et al., 2018), cancer (Paszek et al., 2005; Levental et al., 2009), and liver disease (Nahon et al., 2006).

### Cardiovascular disease

In the context of cardiovascular disease, many studies have supported the idea that changes in arterial stiffness lead to increased atherosclerotic disease and alterations in the physical properties of the arterial wall, which is a biomarker of atherosclerosis (Palombo and Kozakova, 2016). In addition, atherosclerotic plaques have higher stiffness but can cause changes in blood flow, which is associated with worsened disease outcomes (Palombo and Kozakova, 2016). Therefore, these mechanical changes in the environment could modulate the activity and phenotype of macrophages and DCs. Stiffness also influences foam cell proliferation under both homeostatic and inflammatory conditions, as primary human macrophages cultured on softer 1 kPa substrates demonstrated a marked increase in uptake of low-density lipoproteins and oxidized low-density lipoproteins compared to stiffer substrates (Ammanamanchi et al., 2021).

In macrophages, deficiency in Mst1/2 worsens cardiac dysfunction after myocardial infarction, as mice lacking Mst1/2 in their macrophages exhibited a marked increase in left ventricular end-diastolic and end-systolic volume and decrease in ejection fraction and fractional shortening (Liu et al., 2021). In addition, the pharmacological inhibition of YAP/TAZ dampened pro-inflammatory gene expression (IL-1β and IL-12β) (Mia et al., 2020). Additionally, mice with macrophage-specific activation of YAP develop higher levels of cardiac fibrosis and reduced cardiac function following myocardial infarction, compared to wild-type control mice, suggesting that YAP activation in macrophages contributes to increased fibrosis and accumulation of ECM proteins, consequently leading to worsened outcomes following myocardial infarction (Mia et al., 2020).

### Inflammatory bowel disease

In terms of IBD, the effective stiffness of resected bowel portions from patients is significantly higher than healthy controls, which was associated with increased expression of collagen type I genes (Stewart et al., 2018). In addition, large numbers of macrophages are present in colon samples from IBD patients, which can regulate the initiation and resolution of inflammation (Krausgruber et al., 2011). Recent studies have shed light on a role of YAP/TAZ and Mst1/2 in macrophages and DCs in the setting of bowel inflammation, such as during intestinal infections and IBD. YAP regulates PIM/PRM balance, and YAP deficiency in macrophages mitigates dextran sulfate sodium (DSS)-induced colitis (Zhou et al., 2019). Mice lacking Mst1/2 expression in DCs that were infected with ovalbumin-expressing *Listeria* monocytogenes (LM-OVA) exhibited reduced CD8^+^ T-cell responses (Du et al., 2018), suggesting an important role for Mst1/2 in clearance of infections.

The Piezo1 pathway has also been implicated in this setting. Deficiency of Piezo1 in monocytes, macrophages and granulocytes made mice less susceptible to DSS-induced colitis, and treatment with Yoda1, an agonist of Piezo1, exacerbated colitis (Leng et al., 2022). The mechanical signals that trigger these pathways *in situ* are not well understood. Apart from increased stiffness, altered mucus secretion and gut motility could be additional relevant biophysical cues, whose impact on immune function awaits a closer examination.

### Cancer

Cancers are also associated with altered tissue stiffness, as there is an increase in collagen deposition, altered organization of the ECM resulting in elevated intratumoural matrix stiffness (Pickup et al., 2014). This biophysical alteration in the tumour microenvironment promotes tumour cell proliferation and invasiveness, leading to worsened cancer progression and metastasis (Pickup et al., 2014). With increased ECM stiffness and the presence of tumour-associated macrophages, tumour cells up-regulate the expression of epithelial-to-mesenchymal transition-related markers (Alonso-Nocelo et al., 2018). The matrix stiffness potentially polarizes the tumour-associated macrophages and leads them to produce soluble cues and promote a mesenchymal phenotype in tumour cells (Alonso-Nocelo et al., 2018). In addition, increased stiffness of the ECM and the tumor microenvironment can physically block immune cell invasion and lead to impaired anti-tumor responses (reviewed in greater detail elsewhere (Leight et al., 2017)).

Hippo signalling mediators Mst1/2 and YAP/TAZ are important modulators of macrophage and DC function and phenotype that can affect cancer outcomes. After implantation of MC38 colon adenocarcinoma cells, mice with DC-specific deficiency in Mst1/2 showed a striking increase in tumour growth compared to wildtype mice, which was associated with a significantly decreased expression of IFN-γ in CD8^+^ T cells (Du et al., 2018). Interestingly, genetic or pharmacologic inhibition of YAP suppressed tumorigenesis in a THP-1 and colon cancer cell co-culture model (Huang et al., 2017). Inhibition of YAP in differentiating THP-1 cells alone led to decreased PRM marker gene expression without affecting PIM polarization, suggesting an anti-inflammatory role of Yap in this setting (Huang et al., 2017). On the other hand, when both tumors and THP-1 cells are both present in culture, Yap-1 silencing suppressed oncogenic pathways as well as tumor infiltration by tumor-associated macrophages (Huang et al., 2017). We observed that TAZ was crucial for optimal DC function *in vivo,* and a knockdown in TAZ expression in DCs led to impaired T cell-mediated destruction of antigen-bearing tumour cells (Chakraborty et al., 2021). Overall, these observations show that Mst1/2 and YAP/TAZ dysregulation in macrophages and DCs could significantly impact disease outcomes in a context-dependent manner, both directly and indirectly through cross-talk to target tissues.

### Liver disease

Another disease setting with notable stiffness changes is chronic liver disease. Non-alcoholic fatty liver disease (NAFLD) and hepatitis C infection are commonly associated with an increase in fibrosis, which can eventually lead to liver cirrhosis and the development of hepatocellular carcinoma (Khullar and Firpi, 2015; Younossi et al., 2018). Although it is clear that Kupffer cells (KCs), the tissue-resident macrophages in the liver, play an important role in the progression of NAFLD by secreting pro-inflammatory cytokines (Baffy, 2009), the impact of biophysical changes on macrophages and DCs throughout the progression of NAFLD and other liver diseases is unclear.

Recent studies suggest that YAP is a key regulator of macrophage function in the pathogenesis of liver disease. Increased activation of YAP in KCs enhanced the production of pro-inflammatory cytokines and contribute to the development of non-alcoholic steatohepatitis (NASH) (Song et al., 2020). Mice that were deficient in YAP in macrophages/monocytes and fed on a high-fat diet (HFD) had lower levels of hepatic inflammation and improved liver function (Song et al., 2020) compared to HFD-fed wild-type mice. Administration of verteporfin, an inhibitor of YAP that increases levels of 14-3-3a which sequesters YAP in the cytoplasm and targets it for degradation in the proteasome (Wang et al., 2016), to HFD-fed mice, helps to reduce liver inflammation and mitigate the pathogenesis of NASH (Song et al., 2020). Although these studies support the hypothesis that YAP in macrophages worsens the development of liver diseases, there remains a paucity of studies focusing on how mechanosensing pathways can alter DC function during liver diseases, as well as how other mechanotransduction pathways may be involved.

Overall, mechanotransduction pathways can modulate physiological and pathological processes, and this could be mediated by DC and macrophages. Multiple studies support a pathogenic role of Hippo signalling mediators MST1/2 and YAP/TAZ in myeloid cell types. There is also emerging evidence that implicates a role for PIEZO1 in exacerbating lung diseases, as PIEZO1 in macrophages drives autoinflammatory disease pathology in a bleomycin-induced pulmonary fibrosis model (Solis et al., 2019). However, in an *in vivo* disease setting, mechanical and inflammatory signals act simultaneously, making it difficult to dissociate one from the other. Similarly, the mechanical signal transduction pathways sense a variety of inputs. For instance, the activation of the Hippo signalling pathway, which involves mediators Mst1/2 and YAP/TAZ, can be modulated by additional environmental and biological cues including alterations in nutrient levels and cellular polarity (Totaro et al., 2018). Therefore, *in vivo* disease settings, alterations in mechanotransduction likely act in concert with additional biochemical and signals to drive the phenotypic differences in macrophages and DCs. Also, while dysregulation of inflammation is known to exacerbate disease pathogenesis, it is difficult to dissociate the impact of disease-related tissue stiffness changes from the pathological pro-inflammatory changes. To this end, recent studies using genetic knockout models have shed new light on the role of mechanosensing pathways in altering macrophage function during pathological conditions. Additionally, *in vitro* proof-of-concept studies demonstrate that mechanical stimuli can indeed modulate macrophage and DC function and metabolism, and that mechanosenitive pathways may serve as actionable targets for therapeutic strategies that dampen inflammatory diseases and pathologies.

## Discussion

Macrophages and DCs are related immune cells that are present throughout the body and receive input through multiple mechanical signals and result in cellular alterations (Figure 2). For macrophages, the effects of various biophysical cues including ECM stiffness, stretch, and flow have been strongly established, albeit with some inconsistencies among multiple studies. Although relatively less investigated, DCs also possess the ability to integrate extracellular mechanical and immune stimuli and adapt their metabolic program to support processes involved in activation, migration, and antigen presentation. We favor a model where during acute inflammation or inflammatory diseases, where tissues undergo changes in physical properties, macrophages and DCs are not only activated by biochemical cues but also primed by mechanical signals (Du et al., 2022). These innate immune cells detect mechanical forces through integrins connected to F-actin and ion channels Piezo1 and TRPV4. Pulling force from the environment triggers F-actin polymerization and cytoskeleton remodeling. Meanwhile, stress on the cell membrane activates Piezo1 and TRPV4 allowing an influx of calcium ions which also facilitate cytoskeleton rearrangement and Rho GTPases activity. Rho GTPases facilitate phagocytosis and migration necessary for inflammatory responses in macrophages and DCs. Cytoskeleton remodeling and force transduction further modulates the Hippo signaling, allowing YAP/TAZ proteins to translocate into the nucleus where they bind with transcription factors and induce metabolic genes supporting pro-inflammatory phenotypes. These mechanotransduction pathways coordinate with pathogen recognition receptors, tuning their sensitivity, to influence immune cell activation and effector functions.

**FIGURE 3:**
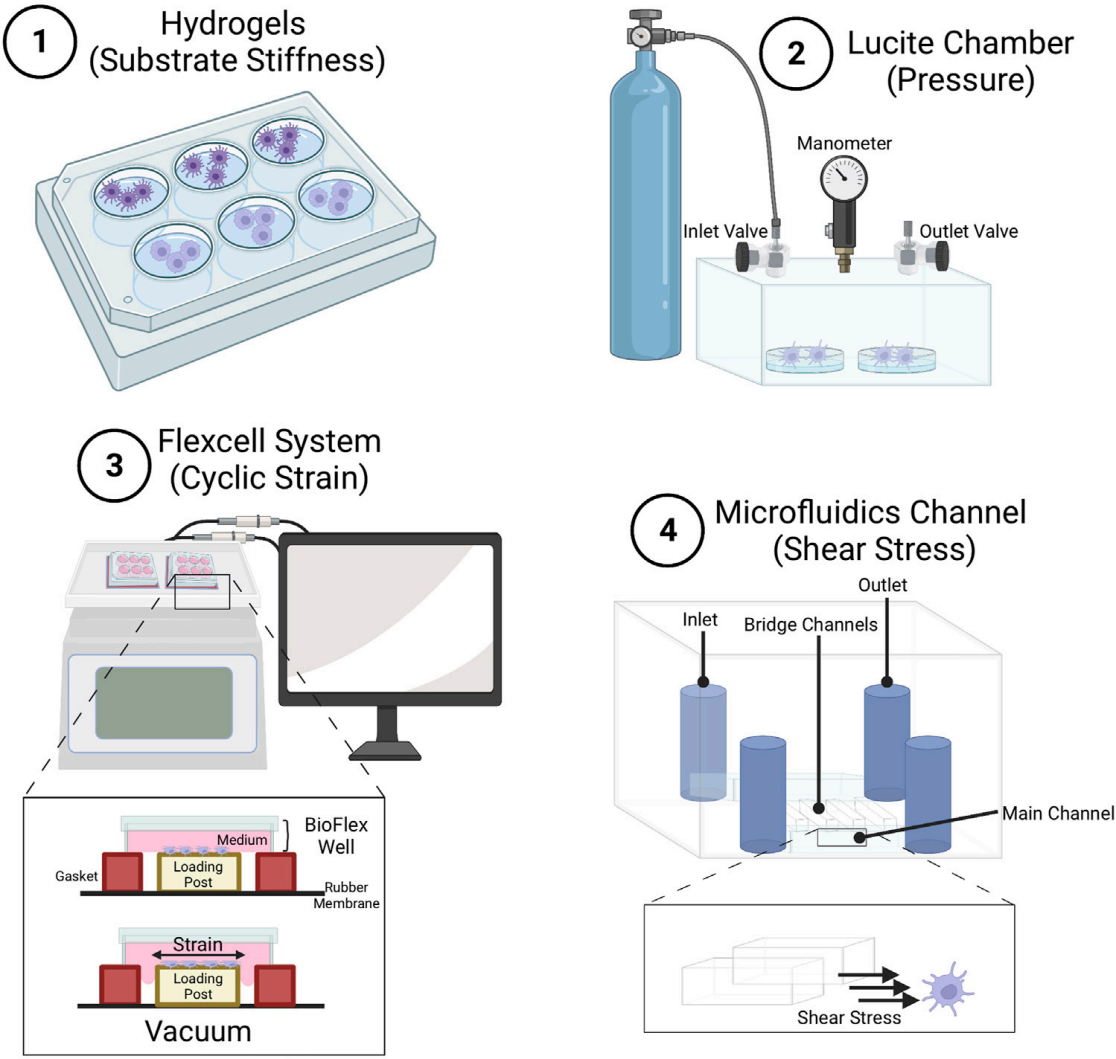
Methods for studying effects of mechanical stimuli on macrophages and dendritic cells. (1) Hydrogels used to study the effect of substrate stiffness of various compliance on immune cell development, differentiation and function. Common hydrogels include poly-dimethyl-siloxane hydrogels (as described in (Lee et al., 2022)), and polyacrylamide hydrogels (Shaheen et al., 2017). Lucite chamber used to apply pressure to cells. Pressure can be controlled using an airtight Lucite box with an inlet valve for gas application, and an outlet valve connected to a manometer. Boxes are prewarmed to 37°C to prevent internal temperature and pressure fluctuations (as described in (Craig et al., 2008)) (Oakes et al., 2009). Flexcell system to apply strain to cells. Cyclic strain can be applied by deformation of the Bioflex well plate through regulated air vacuum supplied to the bottom of the plate, causing the membrane to stretch (Adlerz et al., 2016). Microfluidics channel to apply shear stress on cells. Cells are cultured within the lower main channel, while medium is injected into the upper channel. Cells get exposed to shear stress generated through the bridge channel (as described in (Kang et al., 2021)), and mechanical forces experienced by cells get calculated using computational simulation.

While this model suggests elevated force may promote PRR crosstalk and activation, some other studies show increased inflammation with diminished force inputs. Thus, we favor a system whereby each cell type signals in homeostasis within a defined mechanical force range. Force inputs either too high or too low may be deemed as stressors to ignite inflammatory responses. Understanding the crosstalk between mechanical stimulation and biochemical stimulation of the immune system, as well as ramifications of magnitude and duration of force is an important avenue of future investigation. Another important avenues of future research include the establishment of more *in vivo* models to test such predictions. Although currently there are *in vitro* systems to study different types of mechanical forces (Figure 3), it would also be important to establish newer probes for *in vivo* force measurements as well as *in vitro* culture techniques (Lee et al., 2022) that are readily available to the entire science community to study mechanical force effects in immune cells. While many studies have yielded fascinating insights into the effects of mechanical forces on DC and macrophage function, the field of mechanoimmunology is still in its infancy. Fundamentally, more research, particularly with *in vivo* models, will improve our appreciation of mechanoimmunology, and unravel new therapeutic opportunities to mitigate inflammatory diseases.
